# Geriatric nutritional risk index as a predictor for postoperative complications in patients with solid cancers: a meta-analysis

**DOI:** 10.3389/fonc.2024.1266291

**Published:** 2024-02-07

**Authors:** Weichen Liu, Ming Li, Siqin Lian, Xijie Hou, Ying Ling

**Affiliations:** ^1^ The Department of Blood Purification, First Affiliated Hospital, Guangxi Medical University, Guangxi, China; ^2^ The Department of Oncology, First Affiliated Hospital, Guangxi Medical University, Guangxi, China; ^3^ The Department of Nursing, First Affiliated Hospital, Guangxi Medical University, Guangxi, China

**Keywords:** geriatric nutritional risk index, postoperative complications, solid cancer, CD grades, meta-analysis

## Abstract

**Background:**

The geriatric nutritional risk index (GNRI) has been wildly used to predict the prognosis of patients with solid cancer, but it’s value in postoperative complications remains unclear. The aim of our study was to systematically explore the value of the GNRI in postoperative complications in patients with solid cancer.

**Method:**

The study conducted a systematic literature search using electronic databases to investigate the influence of the GNRI on postoperative complications in patients with solid cancer. The search covered articles published up until May 2023. The odds ratio (OR) with a 95% confidence interval (CI) was employed to assess the effect of GNRI on postoperative complications.

**Result:**

A total of 11 studies with 11,002 patients were enrolled in our meta-analysis. The results suggested that patients with a low GNRI have a higher risk of experiencing postoperative complications (OR=2.51, 95%CI 2.05–3.02, z=9.86, p<0.001), a higher risk of suffering Clavien-Dindo (CD) grades≥2 complications(OR=2.24, 95%CI 1.84–2.73, z=8.01, p<0.001), a higher risk of suffering infection (OR=1.85, 95%CI 1.18–2.88, z=2.70, p=0.007) and a higher risk of suffering respiratory complications(OR = 2.94, 95%CI: 1.56-5.55, z=3.31, p=0.001).

**Conclusion:**

Based on existing evidence, the GNRI was a valuable predictor of postoperative complications in patients with solid cancer.

**Systematic review registration:**

https://www.crd.york.ac.uk/PROSPERO/display_record.php?RecordID=434299, identifier CRD42023434299.

## 1 Introduction

Cancer is a major public health problem worldwide and it has been the second leading cause of death in the United States. According to the 2023 edition of Cancer Statistics, There are 1,958,310 new cases of cancer and 609,820 cancer deaths are projected to take place in the United States (1). It is well known that surgical resection of the lesion is one of the important means to treat early tumor (2), However, the incidence of postoperative complications was high, and the common complications included intestinal obstruction, postoperative bleeding and anastomotic fistula, which significantly affected the prognosis and subsequent treatment of patients (3–5). Studies have shown that the incidence of malnutrition in cancer patients is 20% ~ 70%, and preoperative malnutrition is one of the important factors affecting the occurrence of postoperative complications (6). Malnutrition will not only lead to the increase of postoperative complications, but also prolong the hospital stay, resulting in poor treatment effect and increased mortality (7, 8). Effective nutritional evaluation holds significant importance in managing postoperative complications in tumor patients.

Several nutritional scoring systems have been proposed in previous studies to assess the nutritional status of people with different clinical conditions, such as the dystrophic inflammation score (9), the subjective global assessment (10) and the mini-nutritional assessment (11). However, these parameters are not widely used in clinical practice due to their instability and large differences in baseline values (12). Recently, the Geriatric Nutrition Risk Index (GNRI), a comprehensive malnutrition index that incorporates height, weight, and serum albumin levels, has shown associations with the prognosis and postoperative complications in various types of solid tumors. Such as colorectal cancer (13), esophageal cancer (14) and so on. While several previous meta-analyses have demonstrated the prognostic value of the GNRI in cancer patients, there is a lack of discussion regarding the association between GNRI and postoperative complications in solid tumor patients. This absence of discussion raises certain limitations in the existing literature (15, 16). Therefore, this meta-analysis is based on existing evidence to verify the role of GNRI in postoperative complications in patients with solid tumors.

## 2 Materials and methods

### 2.1 Search strategy

Our meta-analysis adhered to the Preferred Reporting Project for Systematic Review and Meta-Analysis (PRISMA) guidelines. We systematically searched the Web of Science, PubMed, Embase databases, and Cochrane Library to identify relevant literature on the assessment of GNRI in evaluating postoperative complications in patients with solid tumors. The search was conducted up until May 1, 2023. The search was also restricted to English language publications. The search terms and keywords employed included: “GNRI”, “geriatric nutritional risk index”, as well as “cancer”, “carcinoma”, “neoplasm”, and “complication”. In addition, the reference lists of the search literature was reviewed to identify additional potential studies.

### 2.2 Study inclusion and exclusion criteria

The inclusion criteria are shown below (1): Study on GNRI and postoperative complications in elderly cancer patients (2); Cohort or case-control studies (3); In cases where multiple studies by the same author or with overlapping data exist, the study with the largest sample size or the most recent publication date is selected.

### 2.3 Data withdraw and quality assessment

We extracted various variables from each included study, which encompassed the first author’s name, country and year of publication, patient characteristics, study type, sample size, age range, sex ratio, GNRI cut-off point, duration of follow-up, and assessed outcomes. The main endpoint included postoperative complications. The other endpoints included CD grades ≥2, infection, ileus, leakage and respiratory complications.

To evaluate the quality of the eligible studies, two independent investigators utilized the Newcastle-Ottawa Scale (NOS). In instances where there was a disagreement, a third investigator was involved, and consensus was reached through discussion. In our study, a NOS score higher than 7 indicated a high methodological quality.

### 2.4 Statistical analysis

The extracted data were pooled for analysis. An OR > 1 indicated an increased risk of postoperative complications in patients with a low GNRI. Statistical heterogeneity among the included studies was assessed using the I2 statistics. A random-effects model was employed in cases of significant heterogeneity. Subgroup analysis was conducted to evaluate the impact of each subgroup on the combined effect. Sensitivity analysis was performed to assess the stability of the results. Potential publication bias was assessed using Begg’s test and Egger’s test. A significance level of P<0.05 was considered statistically significant. All statistical analyses were conducted using Stata 17.0.

## 3 Results

### 3.1 Literature search

**Figure 1:**
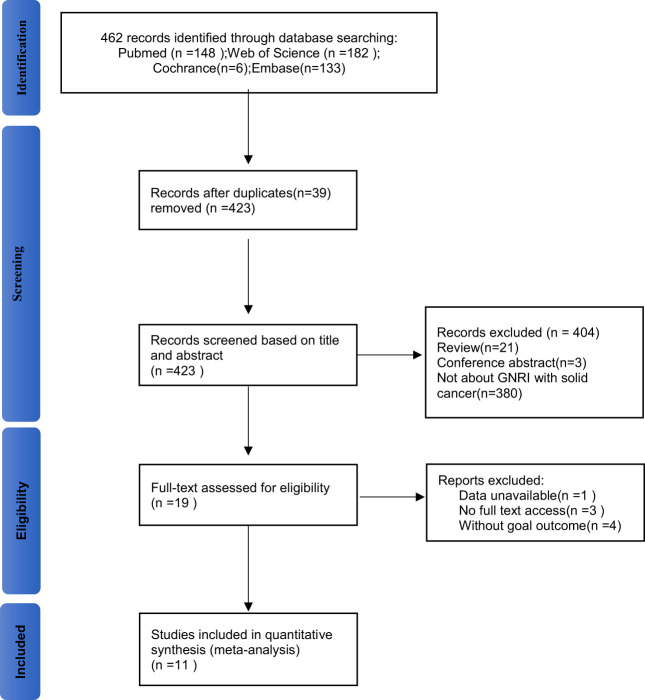
Flowchart of included studies.

**Figure 1** shows the literature screening process of this study. A total of 462 studies were retrieved from the databases according to the search strategy. 39 duplicate studies were removed before screening, leaving 423 studies for further screening. After thorough review of the titles and abstracts, 21 reviews, 3 conference abstracts, and 380 studies that did not focus on GNRI as a predictor of complications in solid cancer patients were excluded. Then, the full texts of the 19 included studies were evaluated. Three of these studies were not able to get the full text; meanwhile, the one with incomplete data was excluded. Four studies were lacked relevant outcomes. Therefore, our meta-analysis included 11 studies involving 11,002patients (8, 14, 17–25).

### 3.2 Study characteristics

The baseline information of the 11 eligible studies is presented in **Table 1**. Out of these studies, 10 were single-center retrospective studies (8, 14, 18–27), while one study was based on a retrospective analysis of a database (17). All of the included studies in this analysis were published between 2015 and 2023. The sample sizes of these studies ranged from 62 to 7863 participants. Six studies were from Japan (14, 19, 22–25), three studies were from China (8, 18, 20), one study from USA (17) and one study from Korea (21). Three studies reported colorectal cancer (8, 20, 23), two studies reported Esophageal cancer (14, 25), two studies reported Gastric cancer (22, 24), two studies reported renal cancer (17, 19), one study reported liver cancer (21) and one study reported prostate cancer (18). In addition, 10 studies reported postoperative complications, six studies reported CD grades. The NOS score for all studies were 8.

**Table 1:** The characteristics of included studies.

No.	Study	Country	No. of Patients	Cut-off of GNRI (no-risk/risk)	Mean/Median Age (years)	Gender Ratio (M/F)	Cancer Location	Surgical Type	Endpoints	NOS
1	Riveros et al. (17)	USA	7863	98 (5750/2113)	71.00 (68.00-76.00)	4948/2915	Renal	Laparoscopic/open (2540/5323)	Complications、CD	8
2	Su et al. (18)	China	96	98 (62/34)	72.50 ± 4.82	96/0	Prostate	Laparoscopic	CDCS	8
3	Watanabe et al. (19)	Japan	62	98 (45/17)	73.1 (65-87)	43/19	Renal	NR	Complications	8
4	Liao et al. (20)	China	1206	98 (544/662)	80.45 ± 4.42	673/573	Colorectal	Open/Laparoscopy (848/358)	Complications	8
5	Lee et al. (21)	Korea	219	98 (162/57)	73.2 ± 5.4	68/151	Liver	Laparoscopic	Complications	8
6	Hirahara et al. (22)	Japan	303	85.7 (272/31)	76 (65-91)	209/94	Gastric	Laparoscopic	Complications	8
7	Tang et al. (8)	China	230	98 (117/113)	70.6 ± 5.4	154/76	Colorectal	Open/Laparoscopy (124/106)	Complications(CD)、OS、FPS	8
8	Sasaki et al. (23)	Japan	313	98 (176/137)	73 (65-94)	201/112	Colorectal	NR	Complications、CD	8
9	Kubo et al. (14)	Japan	240	92 (196/44)	63.4 ± 7.8	193/47	Esophageal	Open/Laparoscopy (80/160)	Complications(CD)、OS、FPS	8
10	Kushiyama et al. (24)	Japan	348	92 (190/158)	79.6 ± 3.8	230/118	Gastric	Open/Laparoscopy (91/257)	Complications、CD	8
11	Yamana et al. (25)	Japan	122	90 (94/28)	63.9 ± 9.1	101/21	Esophageal	Open/Laparoscopy (40/82)	Complications	8

### 3.3 GNRI and postoperative complication

**Figure 2:**
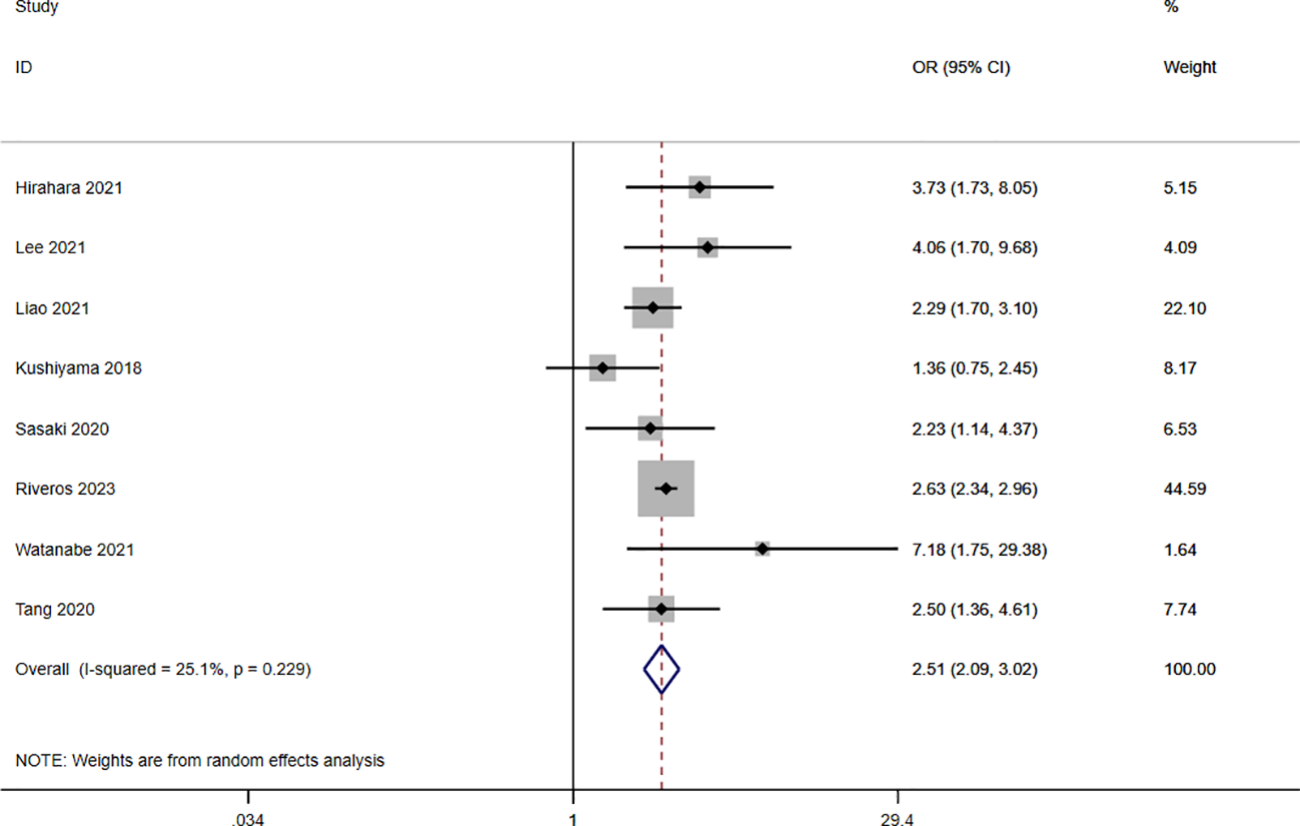
Forest plot for the association between GNRI and postoperative complications.

**Figure 3:**
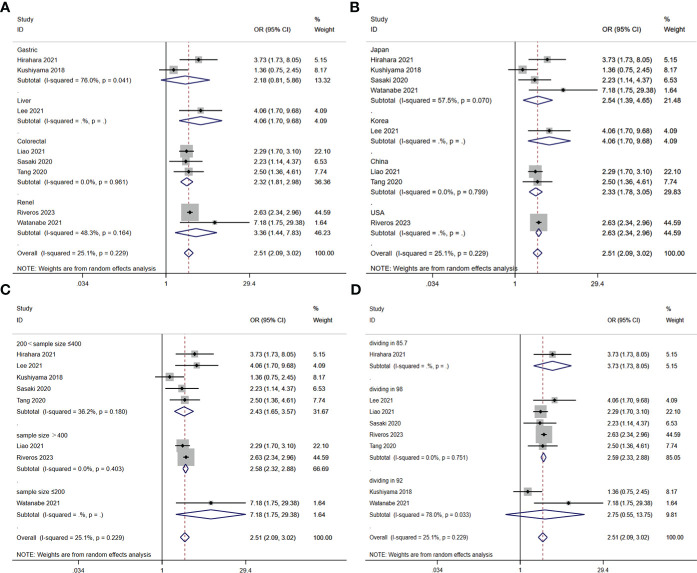
Stratified analysis for the meta-analysis with postoperative complication by cancer site **(A)**, country **(B)**, sample size **(C)**, and cut off value of GNRI **(D)**.

A total of nine studies involving 10,544 patients reported a relationship between the GNRI and postoperative complications in patients with solid cancer. Based on a random effects model (I^2^ = 25.1%, p = 0.229), the GNRI was significantly relevant to postoperative complication (OR=2.51, 95%CI 2.05–3.02, z=9.86, p<0.001) (**Figure 2**). These findings suggest that patients with a low GNRI have a higher risk of experiencing postoperative complications compared to those with a high GNRI. We performed a further subgroup analysis based on cancer site (**Figure 3A**), country (**Figure 3B**), sample size (**Figure 3C**), and cut off value of GNRI (**Figure 3D**). The results revealed that a low GNRI was an independent risk factor affecting postoperative complications in all subgroups. Meanwhile, GNRI was a predictor in liver, colorectal and renal cancer, but have no statistic significant in gastric cancer.

### 3.4 GNRI and CD grades

**Figure 4:**
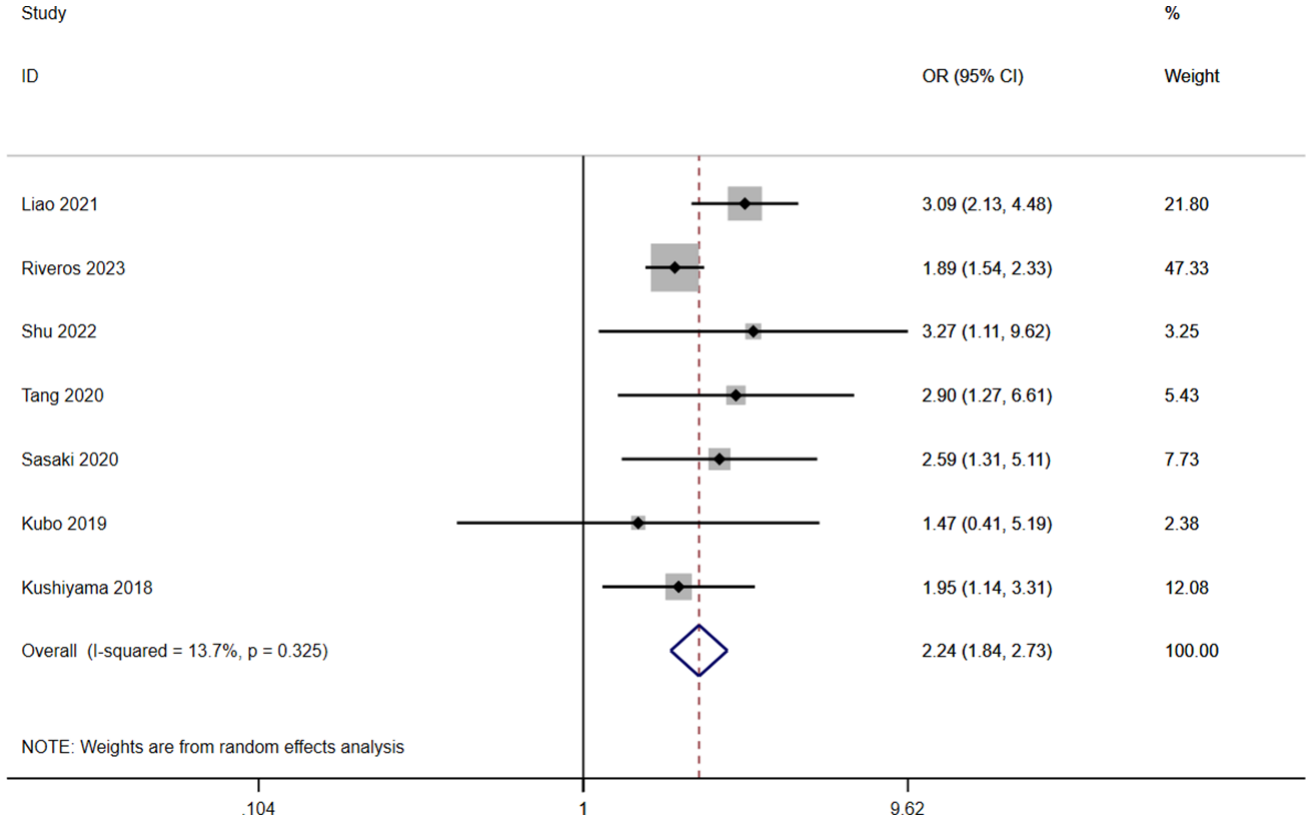
Forest plot for the association between GNRI and CD grades ≥2.

A total of seven studies involving 10,255 patients reported a relationship between the GNRI and CD grades in patients with solid cancer. According to a random effects model (I^2^ = 13.7%, p = 0.325), the GNRI was also significantly relevant to CD grades (OR=2.24, 95%CI 1.84–2.73, z=8.01, p<0.001) (**Figure 4**). It indicated that the patients with a low GNRI had a higher risk of suffering CD grades≥2 complications than those with a high GNRI.

### 3.5 GNRI and infection

**Figure 5:**
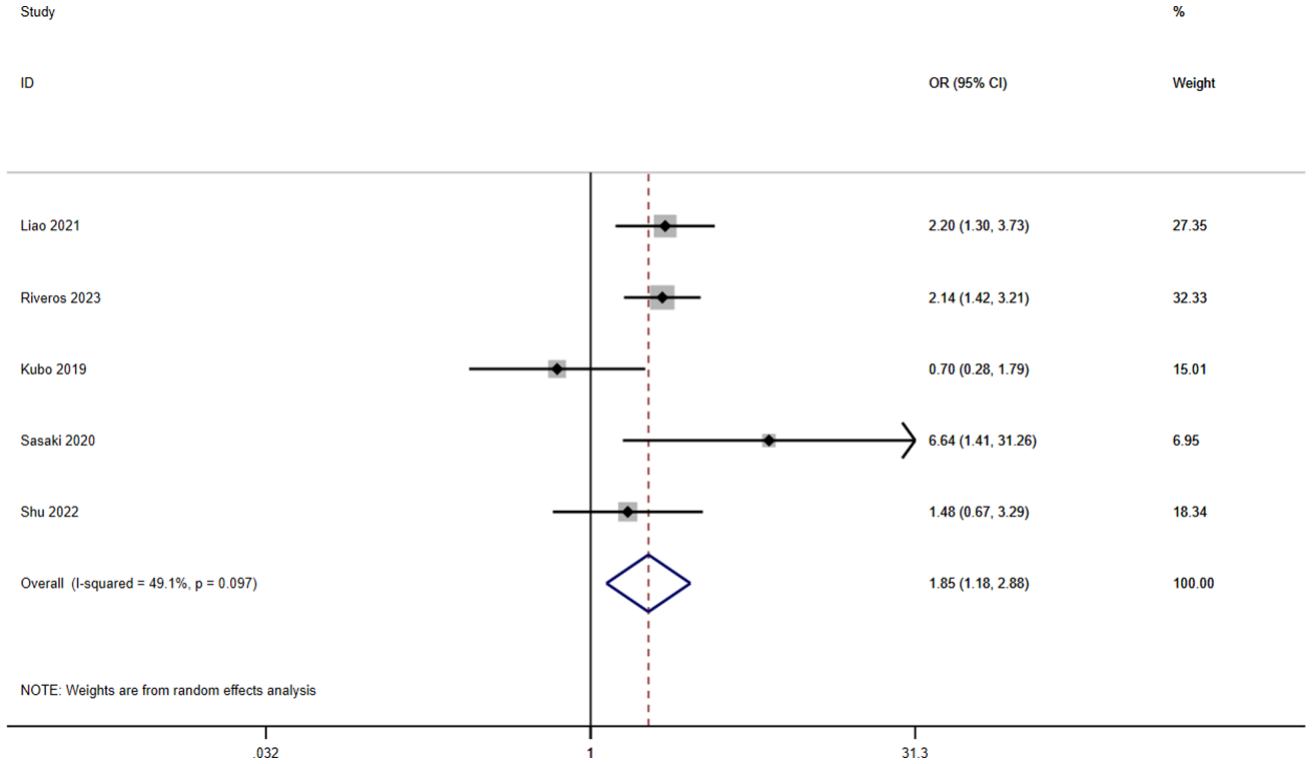
Forest plot for the association between GNRI and infection.

A total of six studies involving 9,718 patients reported a relationship between the GNRI and infection in patients with solid cancer. According to a random effects model (I^2^ = 49.1%, p = 0.097), the GNRI was also significantly relevant to infection (OR=1.85, 95%CI 1.18–2.88, z=2.70, p=0.007) (**Figure 5**). It shows that the patients with a low GNRI had a higher risk of suffering infection than those with a high GNRI.

### 3.6 GNRI and ileus

**Figure 6:**
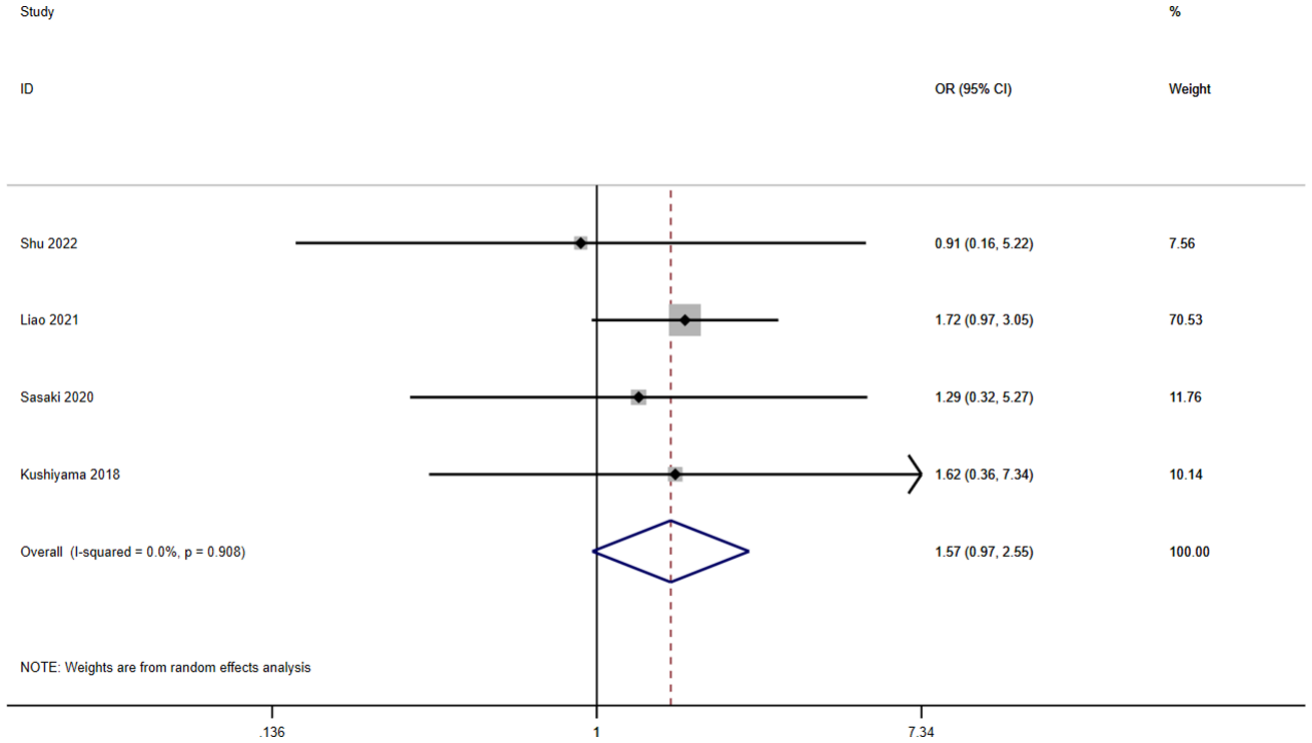
Forest plot for the association between GNRI and ileus.

A total of five studies involving 1,963 patients reported a relationship between the GNRI and ileus in patients with solid cancer. According to a random effects model (I^2^ = 0.0%, p = 0.908), the GNRI had no relevant to ileus (OR=1.57, 95%CI 0.97–2.55, z=1.85, p=0.065) (**Figure 6**).

### 3.7 GNRI and leakage

**Figure 7:**
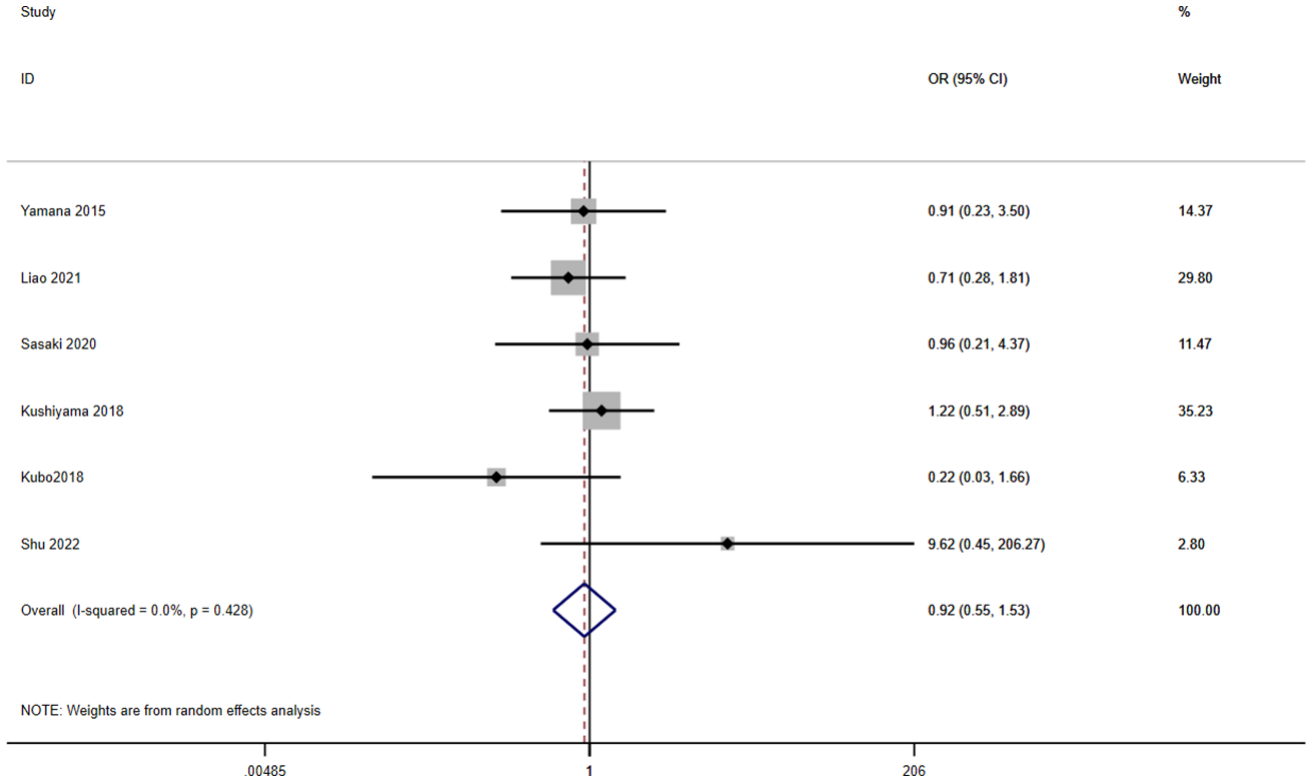
Forest plot for the association between GNRI and leakage.

A total of eight studies involving 2,325 patients reported a relationship between the GNRI and leakage in patients with solid cancer. According to a random effects model (I^2^ = 0.0%, p = 0.428), the GNRI had no relevant to leakage (OR=0.92, 95%CI 0.55–1.53, z=0.33, p=0.741) (**Figure 7**).

### 3.8 GNRI and respiratory complications

**Figure 8:**
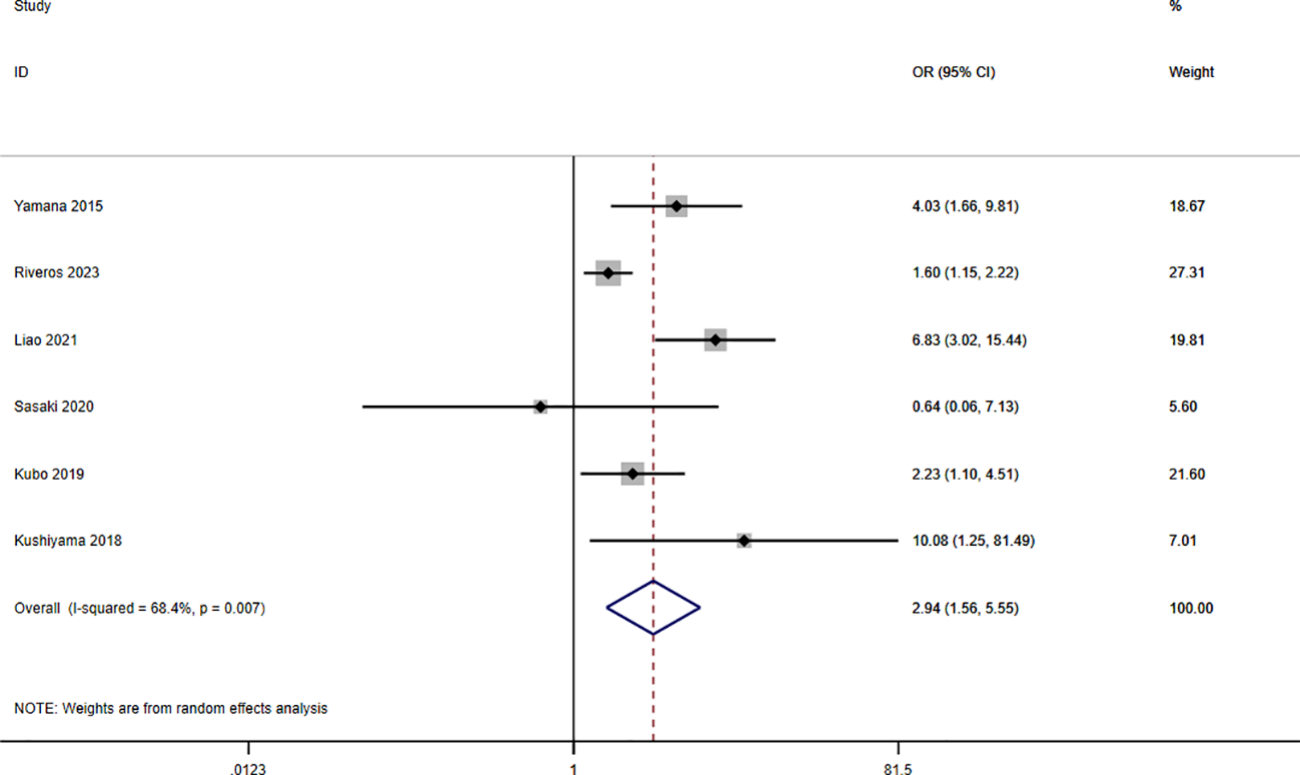
Forest plot for the association between GNRI and respiratory complications.

**Figure 9:**
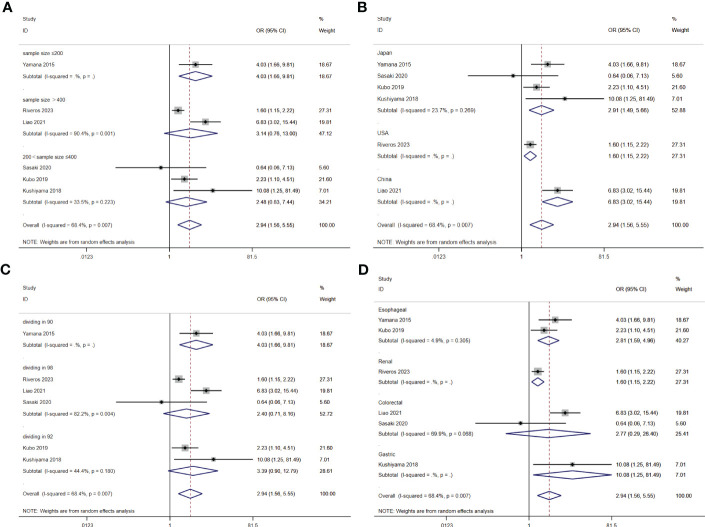
Stratified analysis for the meta-analysis with respiratory complication by sample size **(A)**, country **(B)**, cut off value of GNRI **(C)**, and cancer site **(D)**.

A total of seven studies involving 10,092 patients reported a relationship between the GNRI and respiratory complications in patients with solid cancer. Meta-analysis results from a random effects model (I2 = 68.4%, p = 0.007) evinced that GNRI was also significantly correlated with respiratory complications (OR = 2.94, 95%CI: 1.56-5.55, z=3.31, p=0.001) (**Figure 8**). That is, patients with low GNRI had a higher risk of suffered from respiratory complications than those with a high GNRI. We performed a further subgroup analysis based on sample size (**Figure 9A**), country (**Figure 9B**), cut off value of GNRI (**Figure 9C**), and cancer site (**Figure 9D**). The results revealed that the heterogeneity mainly from the sample size, cut off value and cancer site. It quite stable in country.

### 3.9 Sensitivity analyses

**Figure 10:**
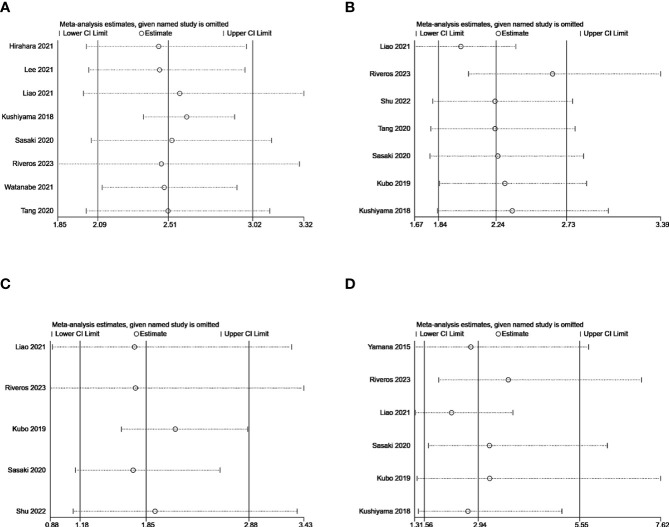
Sensitivity analysis for the correlation of GNRI with postoperative complication **(A)**, CD grades≥2 **(B)**, infection **(C)**, and respiratory complications **(D)**.

Sensitivity analysis was performed to analysis the robustness of our results by removing each individual included study. Omitting any of the included studies did not change the combined meta-analysis effect of GNRI on the ORs for postoperative complication, CD grades≥2, infection or respiratory complications (**Figures 10A–D**. That is to say, our findings were robust across sensitivity analyses.

### 3.10 Publication bias

In the meta-analysis for postoperative complication, no publication bias was found by Begg’s test (p = 0.063), or by Egger’s test (p =0.861). Publication bias was not examined in the other meta-analysis, since the included study number was less than ten.

## 4 Discussion

Geriatric Nutritional Risk Index (GNRI), a novel indicator for evaluating the nutritional risk of elderly medical patients, was first introduced by Bouillanne et al. in 2005 and has been widely adopted since then. The GNRI is a concise and precise screening instrument that relies solely on objective components, such as serum albumin levels, height, and weight measurements. These parameters are readily obtained from laboratory data on a regular basis. The GNRI calculation formula is expressed as follows: GNRI= (1.489 × serum albumin level, g/L) + (41.7 × actual body weight/ideal body weight, kg). GNRI has been proposed as a prognostic factor for various cancers (26). Further studies in oncology have demonstrated that GNRI can also serve as an effective prognostic index for patients with diverse malignancies, not limited to the elderly population (27), but the predictive value of GNRI remains controversial and inconsistent across studies (28, 29). The current literature on the association between GNRI and postoperative complications in solid tumor patients is limited, and the predictive accuracy of GNRI for such complications remains uncertain. Therefore, the objective of this meta-analysis is to investigate the influence of GNRI on postoperative complications in patients with solid tumors. To our knowledge, no previous meta-analysis has been conducted on this subject.

In this study, we included a total of 13 studies involving 12,170 patients with solid cancer. Our findings revealed that GNRI is an independent factor that influences the occurrence of complications in solid cancer patients. Through a stratified meta-analysis, we observed a significant association between low GNRI and postoperative complications, despite variations in country, sample size, GNRI cut-off value, and cancer site among different groups. The consistent results obtained from sensitivity and subgroup analyses underscore the reliability and robustness of our findings. Moreover, there was no evidence of publication bias in the meta-analysis of postoperative complications. Furthermore, we conducted additional investigations to explore the relationship between GNRI and major complications. The results demonstrated that GNRI independently influenced the occurrence of CD grads ≥2, infection, ileus, and respiratory complications in patients with solid cancer. However, no statistical association was observed between low GNRI and leakage.

Based on our study findings, we can conclude that GNRI holds significant clinical value as a practical indicator for predicting postoperative complications in patients with solid cancer. The consistent association between low GNRI and complications such as CD grades ≥2, infection, ileus, and respiratory complications suggests that GNRI can be an effective tool in clinical practice for assessing and managing the risk of postoperative complications in this patient population. Malnutrition is commonly observed in cancer patients and has been linked to both the onset and progression of the disease. Moreover, elderly patients are particularly susceptible to nutritional deficiencies, which can lead to suboptimal treatment outcomes and reduced survival rates (27, 30, 31). Malnutrition can increase the incidence of postoperative complications in cancer patients and have a negative impact on long-term survival. In a study conducted by Shinsuke Kanekiyo et al. (32), it was demonstrated that malnutrition in patients with esophageal cancer undergoing esophagectomy could lead to heightened postoperative complications and mortality. As an index for assessing the nutritional risk of elderly patients, GNRI is primarily linked to body weight and serum albumin levels, which may impact the occurrence of postoperative complications in cancer patients. On one hand, it is well-established that body weight is closely associated with the occurrence of postoperative complications in cancer patients (8). It is well known that our body needs sufficient energy and nutrients to heal after surgery, and inadequate nutrition can weaken the immune system and impair wound healing. However, malnutrition and even cachexia with weight loss as the main manifestation often appear in cancer patients, resulting in poor postoperative prognosis (33). Shu Aoyama et al.’s study suggested that maintaining weight during neoadjuvant chemotherapy may help reduce the risk of postoperative infectious complications (24).This highlights keeping the weight by proper nutrition and support are crucial during cancer treatment to help manage symptoms and improve outcomes. Meredith C Mason et al. found that preoperative weight loss could increase the risk of postoperative complications, including anastomotic leakage (33). Hence, it is obvious that weight loss is an independent factor for adverse prognosis of cancer patients after surgery, which has been proved by O Hynes et al (34) and Bowen Liu et al (35). For another hand, serum albumin level is also strongly correlated with the incidence of postoperative complications (36). Albumin is a protein produced by the liver and is important for maintaining blood volume and transporting various substances in the body, including medications. Numerous studies have demonstrated a positive correlation between perioperative serum albumin levels and patient outcomes. For instance, preoperative hypoalbuminemia is proved to be a predictor of survival (37),or mortality (38).Low preoperative serum albumin levels have been linked to a higher risk of postoperative infections (30), difficulties in wound healing, and prolonged hospital stays (32). Furthermore, Yong Wang et al.’s study discovered that albumin was an independent risk factor for severe complications among colorectal cancer patients following surgery (33). Hence, both weight and albumin levels have a significant influence on the postoperative prognosis of cancer patients. This demonstrates that the GNRI, which is calculated based on body weight and albumin levels, holds predictive value for the occurrence of postoperative complications in solid tumor patients.

In addition to GNRI, commonly used nutritional screening tools for elderly hospitalized patients currently include Subjective Global Assessment (SGA), Mini Nutritional Assessment (MNA), MNA-short form(MNA-SF), Malnutrition Universal Screening Tool (MUST), Simplified Nutritional Appetite Questionnaire (SNAQ), and others (39). SGA is a multidimensional nutritional assessment tool that does not use a numerical scoring system and relies on subjective judgments from professionals, making it less accurate due to variations in different evaluators’ subjectivity (40). MNA has poor specificity for evaluating the nutritional status of hospitalized elderly patients (41, 42) and contains subjective questions that are more suitable for community-living elderly patients (43), potentially leading to overdiagnosis of malnutrition in frail elderly patients and lacking the ability to predict future malnutrition (39). Compared to MNA, MNA-SF requires less than 5 minutes for evaluation and has higher sensitivity and specificity (44). MUST has similar reliability to MNA in screening nutritional risk in elderly populations, with less subjectivity, but research shows that it cannot predict any postoperative clinical outcomes (45). SNAQ is a reliable and effective tool for evaluating appetite loss and weight loss in elderly patients with liver cirrhosis and can be widely used to identify patients at risk of poor appetite and weight loss, but its evaluation parameters focus on appetite (46). Despite controversy surrounding the prognostic ability of albumin, weight, and height used in calculating GNRI, which can be affected by measurement errors, fluid retention, and acute-phase inflammatory reactions (47, 48), GNRI has the advantage of being specifically designed and cross-validated for predicting disease incidence and mortality in elderly patients. It has good predictive validity in design studies and is therefore more suitable for classifying the nutritional status of hospitalized elderly patients and identifying nutrition-related complications (49).

However, it is important to acknowledge the limitations of this study. Firstly, only one included study was a multicenter retrospective study, and the overall number of samples and studies was relatively small. Further investigation through prospective randomized controlled trials is necessary to explore and assess the effectiveness of the GNRI in predicting postoperative complications among patients with solid cancers. Additionally, since the enrolled studies were primarily from Asian countries, the applicability and value of GNRI in other countries still require investigation. Despite these limitations, the available evidence from this meta-analysis confirms the significant predictive value of GNRI for postoperative complications in patients with solid cancers.

## Author contributions

WL: Writing – original draft, Writing – review & editing. ML: Writing – review & editing. SL: Investigation, Writing – original draft. XH: Formal analysis, Investigation, Writing – original draft. YL: Conceptualization, Funding acquisition, Methodology, Writing – review & editing.
